# Abstract of Meteorological Observations Made at Philadelphia, Pa., Latitude 39° 57′ 28″ N., Longitude 75° 10′ 40″ W. from Greenwich, for the Year 1855

**Published:** 1856-02

**Authors:** James A. Kirkpatrick


*Abstract of Meteorological Observations made at Philadelphia, Pa., Latitude* 39° 57′ 28″ *N., Longitude* 75° 10′ 40″ *W. from Greenwich, for the year* 1855. By Prof. James A. Kirkpatrick.

| 1855. | Barometer reduced to 32° F. | | | | | Thermometer. | | | | | Mean Rel. Humidity | | | |
|---|---|---|---|---|---|---|---|---|---|---|---|---|---|---|
| | Monthly Mean. | Highest. | Lowest. | Monthly Range. | Mean Daily Range. | Monthly Mean. | Highest. | Lowest. | Monthly Range. | Mean Daily Range. | Mean. | Highest. | Lowest. | Range. |
| | Inches. | Inches. | Inches. | Inch. | Inch. | Deg. | Deg. | Deg | Deg. | Deg. | Hund | Hund | Hund | Hund |
| January, | 29.971 | 30.610 | 29.163 | 1.447 | .206 | 34.44 | 62 | 16 | 46 | 6.7 | 78 | 100 | 36 | 64 |
| February, | 29.840 | 30.199 | 29.535 | .664 | .159 | 26.67 | 46½ | –1 | 47½ | 5.8 | 71 | 100 | 28 | 72 |
| March, | 29.798 | 30.272 | 29.310 | .962 | .174 | 38.83 | 64 | 16 | 48 | 5.8 | 64 | 92 | 26 | 66 |
| April, | 29.856 | 30.179 | 29.234 | .945 | .165 | 52.88 | 87 | 22 | 65 | 7.1 | 64 | 96 | 24 | 72 |
| May, | 29.807 | 30.124 | 29.449 | .675 | .095 | 63.76 | 87 | 40 | 47 | 5.2 | 57 | 100 | 16 | 84 |
| June, | 29.739 | 30.020 | 29.395 | .625 | .125 | 71.87 | 95 | 51 | 44 | 4.2 | 68 | 94 | 34 | 60 |
| July, | 29.851 | 30.135 | 29.645 | .490 | .086 | 79.71 | 95 | 57 | 38 | 3.9 | 71 | 94 | 43 | 51 |
| August, | 29.923 | 30.255 | 29.514 | .741 | .131 | 75.02 | 84 | 55 | 29 | 3.5 | 69 | 94 | 39 | 55 |
| September, | 29.982 | 30.224 | 29.622 | .601 | .135 | 70.20 | 90 | 45 | 45 | 5.4 | 72 | 94 | 45 | 49 |
| October, | 29.831 | 30.185 | 29.453 | .732 | .131 | 55.22 | 77 | 34 | 43 | 5.8 | 74 | 97 | 42 | 55 |
| November, | 29.948 | 30.228 | 29.330 | .898 | .187 | 48.28 | 67 | 28 | 39 | 5.2 | 71 | 97 | 28 | 69 |
| December, | 29.919 | 30.445 | 28.946 | 1.499 | .261 | 37.50 | 60 | 13 | 47 | 6.4 | 73 | 100 | 40 | 60 |
| Annual Means | 29.872 | 30.6[illegible]0 | 28.946 | 1.664 | .155 | 54.53 | 95 | –1 | 96 | 5.4 | 69 | 100 | 16 | 84 |
| Winter, | 29.899 | 30.610 | 29.163 | 1.447 | .180 | 30.79 | 62 | –1 | 63 | 6.3 | 76 | 100 | 28 | 72 |
| Spring, | 29.820 | 30.272 | 29 234 | 1.038 | .108 | 5 .82 | 87 | 16 | 71 | 6.0 | 62 | 100 | 16 | 84 |
| Summer, | 29.838 | 30.255 | 29.395 | 0.860 | .1i4 | 75.53 | 95 | 51 | 44 | 3.9 | 70 | 94 | 34 | 60 |
| Autumn, | 29.920 | 30.228 | 29.330 | 0.898 | .151 | 57.90 | 90 | 28 | 62 | 5.5 | 72 | 97 | 28 | 69 |
| For 4 years, | 29.908 | 30.709 | 28.895 | 1.804 | | 54.16 | 100½ | –3 | 103½ | | | | | |

| 1855. | Dew Point, 2 P. M. | | | | Rel. Humid., 2 P. M. | | | | Rain & Melted Snow. | Winds. Monthly Resultant. No. of times in 1000. |
|---|---|---|---|---|---|---|---|---|---|---|
| | Mean. | Highest. | Lowest. | Range. | Mean. | Highest. | Lowest. | Range. | | |
| | Degree. | Deg. | Deg. | Deg. | Hund | Hund | Hund | Hund | Inches. | |
| January, | 28.60 | 52.7 | 12.7 | 40.0 | 72 | 96 | 36 | 60 | 2.601 | N. 17¾° W., 464 |
| February, | 18.76 | 45.3 | –2.0 | 47.3 | 71 | 100 | 28 | 72 | 2.480 | N. 50° W., 829 |
| March, | 27.31 | 44.7 | 20.0 | 24.7 | 53 | 92 | 26 | 66 | 1.979 | N, 60° W., 607 |
| April, | 40.45 | 55.3 | 21.3 | 34.0 | 54 | 93 | 24 | 69 | 2.148 | N. 52° W., 395 |
| May, | 46.08 | 60.7 | 25.3 | 35.4 | 43 | 100 | 16 | 84 | 3.033 | N. 48¼° W., 563 |
| June, | 59.37 | 77.5 | 41.3 | 36.2 | 57 | 90 | 34 | 56 | 8.008 | S. 76° W., 458 |
| July, | 68.66 | 78.5 | 52.4 | 26.1 | 62 | 89 | 43 | 46 | 6.594 | [illegible]. 68° W., 429 |
| August, | 64.03 | 75.2 | 47.8 | 27.4 | 62 | 86 | 39 | 47 | 3.237 | N. 33½° W., 078 |
| September, | 60.72 | 71.9 | 43 9 | 28.0 | 61 | 89 | 45 | 44 | 4.129 | N. 67° W., 169 |
| October, | 47.02 | 68.5 | 26.9 | 41.6 | 61 | 97 | 42 | 55 | 3.416 | N. 88½° W., 699 |
| November, | 39.40 | 58.1 | 13.2 | 44.9 | 61 | 97 | 28 | 69 | 2.022 | N. 64° W, 432 |
| December, | 30.05 | 57.3 | 15.1 | 42.2 | 66 | 92 | 40 | 52 | 5.006 | N. 77° W., 475 |
| Annual Means | 44.20 | 78.5 | –2.0 | 80.5 | 6[illegible] | 100 | 16 | 84 | 44.653 | N. 63½* W., 390 |
| Winter, | 24.64 | 52.7 | –2.0 | 54 7 | 72 | 100 | 28 | 72 | 8.266 | N. 45° W., 603 |
| Spring, | 37.95 | 60.7 | 20 0 | 40.7 | 50 | 100 | 16 | 84 | 7.160 | N. 54° W., 521 |
| Summer, | 64.02 | 78.5 | 41.3 | 37 2 | 60 | 90 | 34 | 56 | 17.839 | S. 77° W., 301 |
| Autumn, | 49.05 | 71.9 | 13.2 | 58.7 | 61 | 97 | [illegible]8 | 69 | 9.567 | N. 68° W., 380 |
| For 4 years, | 45 92 | 78.5 | –2.0 | 80.5 | | | | | 44 761 | N. 66¼° W., 365 |

