# Corrections

**Published:** 1856-03

**Authors:** 


CORRECTIONS.—In the General Abstract for 1855, on page 128, the Mean Dew Point for April should have been printed 41.05 instead of 40.45. Also the Resultant of the Winds for October, instead of *N.* should be *S.* 88½° W. 699; and the Mean Daily Range of the Barometer for the Spring Months, instead of .108 should be .145. In the column of Remarks, the *lowest* degree indicated by the Thermometer is printed 1° instead of –1°, or 1 degree *below* zero.

